# Declining Abundance of Beaked Whales (Family Ziphiidae) in the California Current Large Marine Ecosystem

**DOI:** 10.1371/journal.pone.0052770

**Published:** 2013-01-16

**Authors:** Jeffrey E. Moore, Jay P. Barlow

**Affiliations:** Protected Resources Division, Southwest Fisheries Science Center, National Marine Fisheries Service, National Oceanographic and Atmospheric Administration, La Jolla, California, United States of America; Texas A&M University-Corpus Christi, United States of America

## Abstract

Beaked whales are among the most diverse yet least understood groups of marine mammals. A diverse set of mostly anthropogenic threats necessitates improvement in our ability to assess population status for this cryptic group. The Southwest Fisheries Science Center (NOAA) conducted six ship line-transect cetacean abundance surveys in the California Current off the contiguous western United States between 1991 and 2008. We used a Bayesian hidden-process modeling approach to estimate abundance and population trends of beaked whales using sightings data from these surveys. We also compiled records of beaked whale stranding events (3 genera, at least 8 species) on adjacent beaches from 1900 to 2012, to help assess population status of beaked whales in the northern part of the California Current. Bayesian posterior summaries for trend parameters provide strong evidence of declining beaked whale abundance in the study area. The probability of negative trend for Cuvier's beaked whale (*Ziphius cavirostris*) during 1991–2008 was 0.84, with 1991 and 2008 estimates of 10771 (CV = 0.51) and ≈7550 (CV = 0.55), respectively. The probability of decline for *Mesoplodon* spp. (pooled across species) was 0.96, with 1991 and 2008 estimates of 2206 (CV = 0.46) and 811 (CV = 0.65). The mean posterior estimates for average rate of decline were 2.9% and 7.0% per year. There was no evidence of abundance trend for Baird's beaked whale (*Berardius bairdii*), for which annual abundance estimates in the survey area ranged from ≈900 to 1300 (CV≈1.3). Stranding data were consistent with the survey results. Causes of apparent declines are unknown. Direct impacts of fisheries (bycatch) can be ruled out, but impacts of anthropogenic sound (e.g., naval active sonar) and ecosystem change are plausible hypotheses that merit investigation.

## Introduction

**Figure 1:**
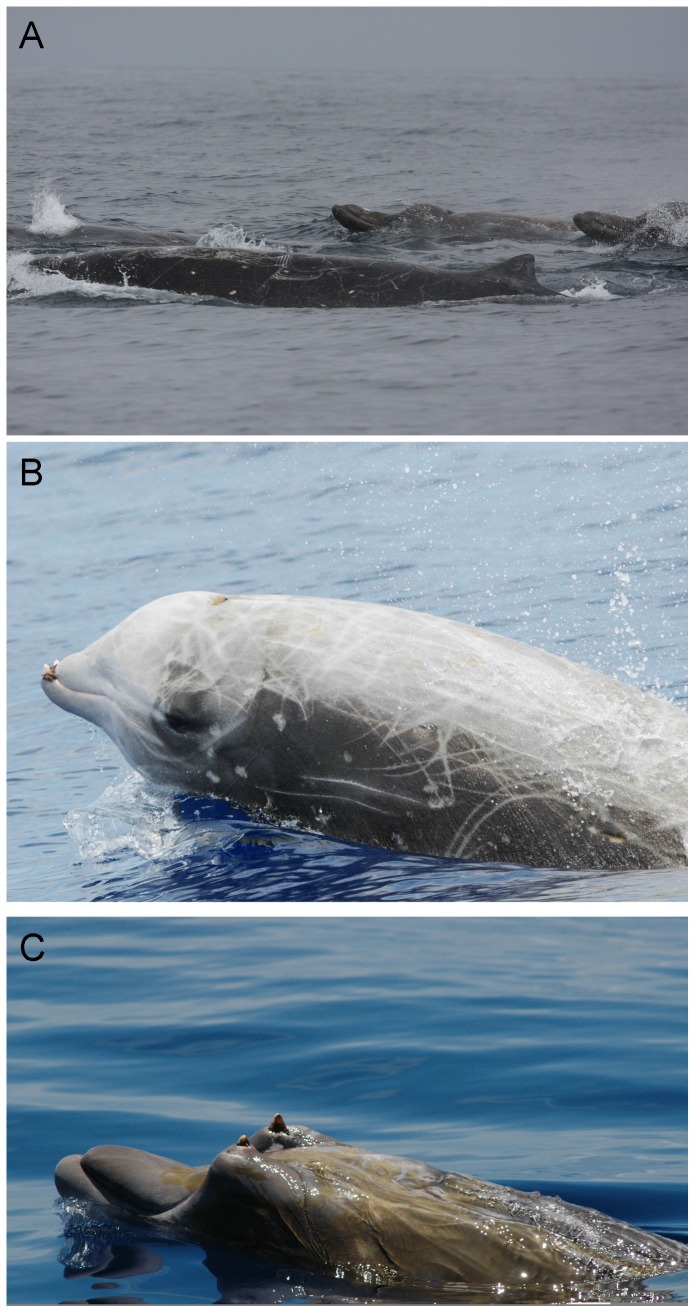
Example species of beaked whales occurring in the California Current Large Marine Ecosystem. (a) group of Baird's beaked whales, *Berardius bairdii*; (b) Cuvier's beaked whale, *Ziphius cavirostris*, the most abundant species in our study system; (c) Blainville's beaked whale, *Mesoplodon densirostris*, a warmer-water species rare in our study area (Photo credits – a: Bob Pitman, SWFSC; b and c: Bahamas Marine Mammal Research Organisation).

Beaked whales (Family Ziphiidae, Fig. 1) are one of the most diverse groups of marine mammals, comprising 21 (24%) of the 87 extant cetacean species currently recognized by the Society for Marine Mammalogy [1]. Among all marine mammal taxa, only the dolphin family (Delphinidae) is more speciose. Yet, the ecology and conservation status of ziphiids are the least understood for all marine mammal groups, owing to their deep-water oceanic existence and typically inconspicuous surface behavior. Feeding in depths often exceeding 1000 m [2], [3], most species are rarely seen; some have never been identified alive at sea and are known only from beach-stranded carcasses [4]. According to the IUCN Red List, approximately 40% of marine mammal species are considered Data Deficient [5], whereas for the Ziphiidae, 90% are Data Deficient (all except Cuvier's beaked whale, *Ziphius cavirostris*, and southern bottlenose whale, *Hyperoodon planifrons*, which are Least Concern). Population trends for all beaked whale species are listed as unknown on the IUCN Red List.

While little is known about beaked whale ecology, and in spite of their cryptic existence, there is nevertheless a long list of documented human impacts to beaked whales [6], [7]. Beaked whales are hunted (mainly Baird's beaked whale, *Berardius bairdii*), entangled unintentionally in fishing nets, affected behaviorally and physiologically by naval active sonar (sometimes with lethal effects), disturbed to an unknown extent by other ocean noise sources such as from large commercial vessels or oil and gas seismic surveys, susceptible to health effects of ingesting plastic debris, and potentially vulnerable to deepwater ecosystem changes driven by climate-related oceanographic forcing or other human impacts such as demersal fishing. Given this diverse set of mainly anthropogenic threats, there is an obvious need for improving our ability to assess beaked whale population status and impacts of anthropogenic activities.

The challenge of assessing abundance trends for rare or cryptic wildlife species is a long- standing problem in ecology [8], [9]. Cetacean abundance trends can be notoriously difficult to estimate based on monitoring programs because of typically low precision associated with individual abundance estimates [10], [11]. However, studies in terrestrial systems first demonstrated the value of using Bayesian hierarchical analyses to improve abundance trend inference by making efficient use of information contained within a time series of replicate-survey or capture-recapture data [12], [13]. Extending these lessons to a distance sampling framework, Moore and Barlow [14] estimated abundance and assessed population trends for fin whales (*Balaenoptera physalus*) from a time series of line-transect survey data. In essence, the problem of small samples from individual surveys can sometimes be overcome by building up a larger sample over the course of multiple surveys, since all the observations provide information about the same Markovian biological process. Thus abundance survey data from one year provide a certain amount of information about population abundance in other years.

**Figure 2:**
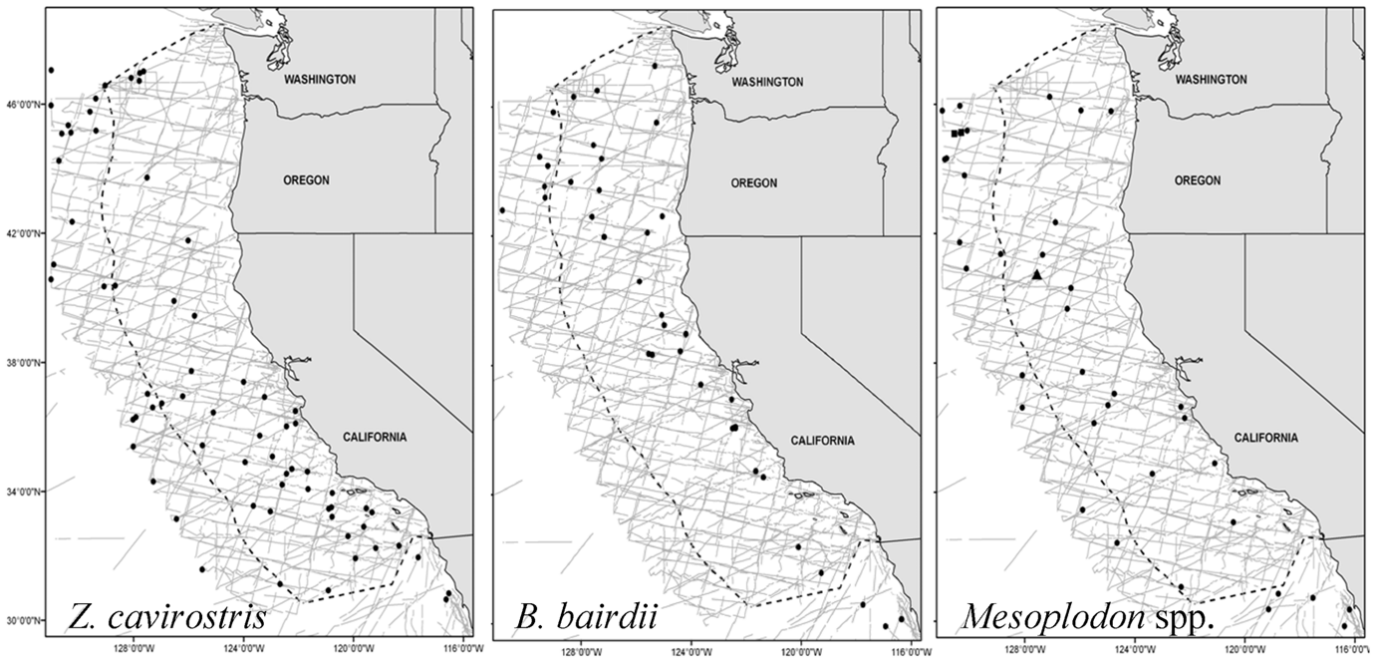
Map of study area, survey effort, and beaked whale sightings. Study area (*A*≈1.142×10^6^ km^2^) demarcated by extent of on-effort transect lines, US EEZ boundary (dotted line), and sighting locations of Cuvier's beaked whale (*Z. cavirostris*), Baird's beaked whale (*B. bairdii*), and *Mesoplodon* spp., from 1991–2008 (from US Marine Mammal Stock assessments [16]).

The NOAA Southwest Fisheries Science Center (SWFSC) has systematically conducted vessel-based visual line-transect surveys for marine mammals in the California Current large marine ecosystem (survey area, *A*≈1.142×10^6^ km^2^) since 1991. Although visual sightings of beaked whales in a given year were relatively few (Table 1), accumulation of sightings over the course of six surveys allowed us to investigate abundance trends. Additionally, records for beach-stranded marine mammals along the western coasts of U.S. and Canada have been archived since ca. 1900 by local and regional stranding networks and museums, providing supplementary information about temporal patterns of beaked whale abundance and distribution. The sum of available information permits an analysis that may help assess conservation status of beaked whales in this part of the California Current. Beaked whale species known to occur in the study area (Figs. 1, 2) include Baird's beaked whale (*Berardius bairdii*), Cuvier's beaked whale (*Ziphius cavirostris*), and at least six species of the genus *Mesoplodon* that cannot be easily distinguished in the field – Hubbs' beaked whale (*M. carlhubbsi*), Blainville's beaked whale (*M. densirostris*), Gingko-toothed beaked whale (*M. gingkodens*), Perrin's beaked whale (*M. perrini*), Pygmy beaked whale (*M. peruvianus*), and Stejneger's beaked whale (*M. stejnegeri*) [15], [16]. Our analysis provides evidence of declining abundance trends for *Z. cavirostris* and for *Mesoplodon* spp. as a pooled group. To our knowledge, these are the first abundance trend assessments published for any beaked whale species. We discuss some plausible hypotheses for the apparent declines.

**Table 1:** Survey data summary.

	1991	1993	1996	2001	2005	2008
*L_t_* (km)	10,025*	6235*	14,674	9537	10,838	11,564
*Berardius bairdii*	2	3	5	2	3	5
*Ziphius cavirostris*	18	12	9	5	3	10
*Mesoplodon* spp.	6	7	15	0	3	1
unidentified ziphiid	0	3	3	2	4	4

Number of beaked whale groups detected, and total km of survey effort (*L_t_*) in each year of cetacean line-transect surveys. Only whale groups <4 km from the transect line, and only survey effort during Beaufort sea state ≤5 are included.

* Effort in these years are from three survey strata only (i.e., Oregon-Washington stratum not surveyed in these years).

## Methods

### Cetacean abundance surveys

Shipboard line-transect surveys for marine mammals were conducted in the California Current by the SWFSC in summer/autumn of 1991, 1993, 1996, 2001, 2005, and 2008 (Fig. 2). The study area has been consistently divided into four strata from north to south: Oregon-Washington, Northern California, Central California, and Southern California. However, because of small sample sizes for beaked whales, the survey strata were collapsed into a single large study area for this analysis. Waters off Oregon and Washington (≈28% of the study area) were not surveyed in 1991 or 1993 (implications of this discussed in Results – Sensitivity analysis). Transects followed a uniform grid pattern anchored to a different random starting point each survey year. Two observers each used mounted 25× binoculars to search for cetaceans from the flying bridge (≈15 m above the water surface) of NOAA research vessels. The vessels closed on all sightings to record group size and confirm species identification. For each sighting, group size was estimated, and a perpendicular distance to the transect line was calculated from the estimated radial distance and a measured sighting angle; various covariates associated with each detection were recorded (e.g., various visibility measures, environmental conditions). These survey methods have been used by many NOAA survey cruises in different areas of the Pacific and are well documented in the literature; for additional details, see [17], [18].

Detections and effort occurring during sea state conditions of Beaufort 0–5 were included in the analysis (although there were no detections in Beaufort 0 conditions, which rarely occur in the region). Distance data were truncated to only include observations <4 km from the transect line; this eliminated 17% (4 of 24) of *B. bairdii* groups and 6% (7 of 112) of groups of *Z. cavirostris*, *Mesoplodon*, and unidentified beaked whales (which belonged to either *Z. cavirostris* or *Mesoplodon*). These data truncations are consistent with recommendations for distance sampling analysis [19]. Total survey effort (on-effort transect length) and counts of beaked whale groups in the full study area are summarized in Table 1.

### Analytical methods for survey data

Analytical methods generally follow those described in our previous analysis of fin whale abundance trends [14]. A brief description is provided here.

#### Process and Observation Models

Models were developed separately for *B. bairdii*, *Z. cavirostris*, a single *Mesoplodon* species group, and a group of unidentified ziphiids (which were either *Z. cavirostris* or *Mesoplodon*), although sighting-distance data were pooled across these groups for purposes of estimating parameters of the detection function. Recognizing that *B. bairdii* are more easily detectable than other ziphiids (they are larger, occur in larger groups, and have more conspicuous blows and surface behavior), the detection model included covariates for inter-species differences (see below).

Following [14], the model for each species group is partitioned into process and observation components. The process model describes how animal density (*D*) changes through time, so that abundance at time *t*, *N_t_* = *D_t_* * *A*, where *A* is the size of the study area. The most general model we considered describes variation in animal density simply as a function of a single temporal trend parameter (*β*_1_) and a stochastic error component (random variable, *γ_t_*), for each year (*t*). Small sample sizes precluded more complex (e.g., geographically stratified) models. If the population is changing exponentially, the full density model is:
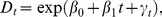
(1)

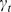
∼Normal(0, *σ*).

The observation model links the state process to the observed data. Following line-transect sampling theory [19], and treating the observed counts of groups each year as a Poisson random variable [14]:
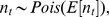


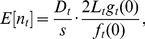
(2)where *n_t_* is number of groups detected; *s* is a single mean group size estimate for the species (there was no evidence of annual variation) with overdispersed Poisson variance (see [14]); *f_t_*(0) is the value at distance *y* = 0 of *f_t_*(*y*), which is the pdf of the detection probability function *g_t_*(*y*), with *g_t_*(0) being the detection probability on the transect line; and *L_t_* is the on-effort transect length (km), considered to be measured without error (Table 1). If variance in the observed counts is over-dispersed (i.e., extra-Poisson), this should be handled implicitly by the process error term in equation 1. This can be seen by substituting the expression for *D_t_* (eqn 1) into equation 2 and re-arranging slightly so that the error term, *γ_t_*, moves outside of the density term:
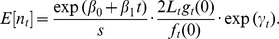
Thus, we may think of *γ_t_* as the sum of *γ_t_*_,p_+*γ_t_*_,s+_, where subscripts p and s+ refer to process error and extra-Poisson sampling error, respectively. Estimates of *γ_t_*_,s+_ in individual years from bootstrapping methods (e.g., [18]) could potentially be used to obtain more explicit estimates of process variance; this would be useful for projecting future abundance estimates with greater precision.

A more intuitive expression of equation 2 is:
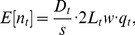
(3)where *w* equals the data truncation distance (4 km in our case) and *q_t_* is the average detection probability of a group within the surveyed area 2*L_t_w*. Equation 3 thus indicates that the expected number of groups detected equals the group density, multiplied by the area surveyed and the average detection probability within the area surveyed, defined as *q_t_* = *g_t_*(0)/*f_t_*(0)·1/*w*. In other words, *q_t_* is the “effective strip half-width” [1/*f_t_*(0)] divided by the total distance from the vessel within which searching takes place and corrected for imperfect detection on the trackline. The effective strip half-width is a mathematical re-interpretation of the distance function *g_t_*(*y*) into a single theoretical distance from the transect line within which groups have a detection probability of 1 and beyond which the probability is zero.

Detection probability decreases as Beaufort sea state increases. Thus the estimate of *q_t_* in equation 3 is:
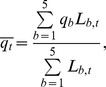
where *L_b,t_* is the amount of survey effort in each of five Beaufort categories (*b* = 1 (for classes 0 and 1), 2, …5) in year *t*, and *q_b_* = *g_b_*(0)/*f_b_*(0)·1/*w*. Note, the estimate for 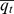 is calculated from the effort-weighted mean of the ratio [*g_b,t_*(0)/*f_b,t_*(0)], not the ratio of the means 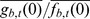. Based on previous analyses in our case study system [18] we assume a half-normal detection function for *g_b_*(*y*):
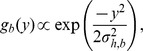
where *h* denotes half-normal parameters and the proportionality sign is used since *g*(0) may be less than 1. We estimated the scale parameter *σ_h,b_* and hence *f_b_*(0) as a function of covariates [20], assuming the following model:
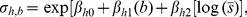
(4)where *β_h_*_0_ is the intercept; and *β_h_*_1_ and *β_h_*_2_ are the coefficients for Beaufort sea state and the log of mean group size for the species, respectively (we use log of group size following convention of earlier SWFSC cetacean abundance analyses [14], [18]). The covariate model is based on the one used by Barlow and Forney [18] for beaked whales, the main difference being that we did not include a categorical variable for the ship on which observations occurred. Preliminary analyses did not reveal this variable to have much importance on parameter estimates, while it complicated the weighted-mean estimation of 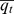. Species group (*B. bairdii* vs. other/smaller species) was initially considered as a covariate as well (and was included in a Sensitivity analysis – see Results), but the sample size for *B. bairdii* was small (Table 1); preliminary analyses suggested that group size was a more useful variable overall and sufficiently acted as a proxy for *B. bairdii* since they usually occur in larger groups. As sample sizes for *B. bairdii* increase with future surveys, a separate variable for them should be included. The parameters for equation 4 were estimated from data for individual detections:
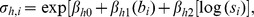
where *i* denotes each observed group (all species detections pooled).

Trackline detectability, *g_b_*(0), for *Z. cavirostris* and *Mesoplodon* beaked whales declines strongly with deteriorating Beaufort sea state conditions. Barlow and Forney [18] reported estimates of *g_b_*(0) for Beaufort states 0–1 (from [21]); these account for the combination of perception bias and availability bias. Estimates for these and Beaufort 2–5 (Barlow, unpublished data) are included in Table 2. The CVs of the *g_b_*(0) estimates are based on Beaufort 0 and 1 conditions [21]; this CV was used for the other sea state levels as well as there are no independent CV estimates available for states 2 and higher.

**Table 2:** Estimates of trackline detection probability, *g*(0).

Beaufort sea state	*Berardius*	*Ziphius*	*Mesoplodon*
0 & 1	0.87*	0.230	0.450
2	0.87*	0.148	0.290
3	0.87*	0.110	0.215
4	0.87*	0.043	0.085
5	0.87*	0.024	0.048
CV	0.23	0.35	0.23

Estimates for each beaked whale genus are a function of Beaufort sea state. Estimates for sea state 0–1 and CV are from [21]. Estimates for *Ziphius and Mesoplodon* in sea states 2+ are from unpublished data (J. Barlow).

* Barlow (1999) reported a point estimate of 0.96. Value reported here is the mean of Barlow's bootstrap distribution, for compatibility with the reported CV.

#### Parameter Estimation

Parameter estimation was conducted using a Bayesian MCMC approach in WinBUGS 1.4.3 [22], [23]. Likelihoods were Poisson for the *n_t_* data, overdispersed Poisson for group size (*s_i_*) data, and truncated half-normal for the distance (*y_i_*) data. See Appendices S1 and S2 in Moore and Barlow [14] for example WinBUGS code and likelihood expressions. Vague priors were used on all parameters except for *g_b_*(0). Informative Beta priors were used for *g*_1_(0) corresponding to Beaufort 0 and 1 estimates in Table 2. At each MCMC sample, g(0) for the other sea state levels were calculated by multiplying *g*_1_(0) by a constant to preserve the *g*_1_(0)∶*g_b_*(0) ratios in Table 2. Normal priors with mean = 0 and large variance (e.g. 10,000) were used for most intercept and slope coefficients (e.g. *β*'s). Positive uniform distributions (e.g., U[0, 10]) were used for standard deviations of random effects. For each model, MCMC runs consisted of two chains with a burn-in of 25,000 samples and a posterior distribution based on 75,000 samples for each chain thinned by 4 (i.e., posterior distributions constructed from 37,500 samples total); this was sufficient to achieve low Monte Carlo errors (<5% of MCMC sample standard deviation) and 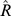
≈1 for key parameters.

#### Abundance of “unidentified beaked whales”

The abundance of the “unidentified ziphiid” group was modeled as a separate species, but these animals are believed to belong to either *Mesoplodon* or *Z. cavirostris*. Adult *B. bairdii* are larger and with distinctive blows that make them unlikely to be confused with the smaller genera. Therefore, *q_t_* for the unknown group was estimated as a weighted average of *q_t_* for *Mesoplodon* and *Z. cavirostris*, with weights at each MCMC sample given by the posterior estimates of relative abundance for these two groups. These weights were also used to proportionally attribute abundance estimates for the unidentified group to *Mesoplodon* and *Z. cavirostris*, thus providing revised estimates of annual abundance and trends.

### Strandings data

Beaked whale stranding records (species, dates, and locations) dating back to 1900 were compiled from two sources: museum collections and four U.S. and Canada west coast regional stranding networks (Alaska, British Columbia, Washington-Oregon, and California). Museum records were accessed mostly through MaNIS (http://manisnet.org) and Arctos (http://arctos.database.museum/home.cfm) internet data portals in July 2012 (see Table S1 for stranding data summaries and sources). Collections managers from all museums verified that these databases accurately reflected museum inventories at date of download. Regional stranding networks developed formally in the early and mid-1980s, although some of them opportunistically collected earlier records as well. There is redundancy between records from the two source types (some animals recorded by stranding networks also reside in museums); therefore, the data sets were evaluated separately, rather than combined into a single data set. These data sources do not comprise *all* known historical stranding records, but we consider them the most representative for describing large-scale spatio-temporal reporting patterns. Some formal stranding networks with a more local focus were established prior to regional networks (e.g., [24]), and earlier records exist from a variety of sources [15], though many of these are included in the museum or stranding network record as well.

Spatio-temporal stranding patterns can provide information about species abundance and distribution, but they also reflect patterns in detection rates, variation in ocean currents that carry carcasses to shore, changes in mortality rates, and other unquantified factors that limit ecological inference. Therefore, we did not attempt formal analysis of strandings data but simply looked for qualitative patterns that seemed obviously consistent or inconsistent with survey results. We limited our evaluation to unique stranding events rather than total numbers of stranded individuals. Thus, multiple individuals stranding together or in nearby locations (within ≈2 degrees latitude or longitude) within a one-week period constituted one event for purposes of generating and visually assessing data plots.

## Results

### Group size and detection

Mean (SD) of the Bayesian posterior distributions for group sizes (*s*) across all surveys were 9.6 (8.7) for *B. bairdii*, 1.81 (0.13) for *Z. cavirostris*, 1.77 (0.17) for *Mesoplodon*, and 1.51 (0.20) for unidentified ziphiids. The smaller mean group size for the unidentified ziphiids may indicate that smaller groups in the field are less likely to be identified, or that groups not seen well enough to identify also tend to be underestimated in size, or the difference could be due to chance. The estimates for *Mesoplodon* and *Z. cavirostris* were slightly lower than the average of previously reported estimates using data from the same surveys [18], [25]. For both groups, the mean group size in our full dataset (Beaufort 0–5 observations) was approximately 1.8, compared to 2.0 and 2.2 for *Mesoplodon* and *Ziphius*, respectively, in the earlier studies, which used Beaufort 0–2 observations only. Differences could reflect sampling error, since we estimated group size from a larger dataset. Alternatively, group size estimates recorded in rougher seas could be biased low, driving down our estimates, although a *post hoc* linear regression of group size vs. Beaufort sea state suggested this possible bias only for the *Z. cavirostris* data.

**Figure 3:**
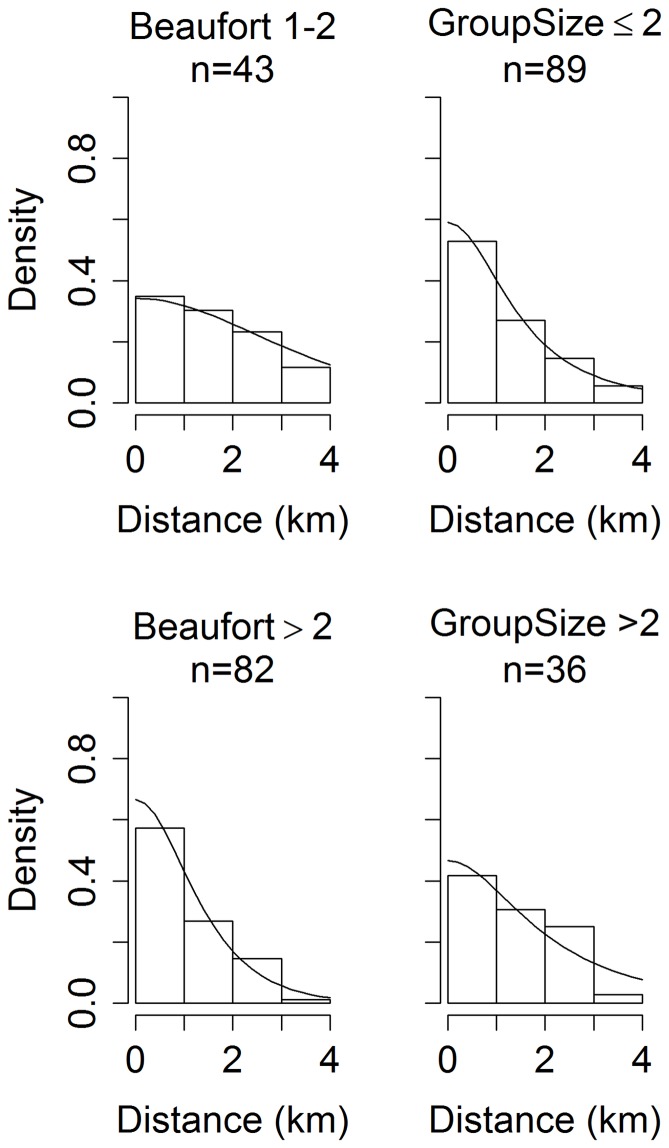
Summary of beaked whale detection distances and detection function. Histograms of beaked whale group detection distances and mean probability density curve, *f*(*y*), of the observations, based on coefficient estimates in (Table 3). Plots are shown for observations in calm (Beaufort 1–2) and rough (Beaufort 3+) sea state conditions, and for small (1 or 2 individuals) and larger (3+ individuals) groups.

**Figure 4:**
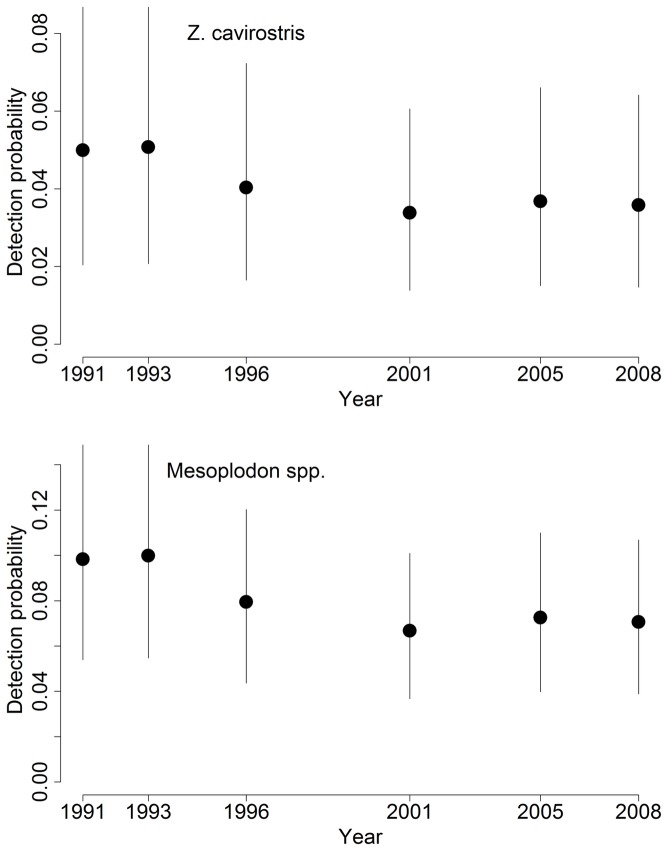
Detection probability through time. Average detection probability of Cuvier's beaked whale and *Mesoplodon* groups occurring within the truncation distance (4 km) of the research vessel. Plotted values are the medians and 95% CRI of the Bayesian posterior distributions.

Detection probability, *g*(*y*), decreased strongly as Beaufort sea state level increased and appeared to increase some with group size, as indicated by posterior distributions for detection model coefficients in the model for *σ_h,b_* (Table 3, Fig. 3). Average sea-state conditions and thus detection probability estimates for *Z. cavirostris* and *Mesoplodon* declined over the course of the study, and the average probability (*q_t_*) of detecting a *Ziphius* or *Mesoplodon* group present within the 4-km truncation distance from the vessel was 0.03–0.05 (CV≈0.36) and 0.07–0.10 (CV≈0.25), respectively (Fig. 4). For *B. bairdii*, estimates of average detection probability declined slightly over the course of the study (due to trend in *f_t_*(0) but not *g_t_*(0)) from 0.49 in 1991 to 0.43 in 2008 (CV≈0.27).

**Table 3:** Posterior distribution summaries for coefficients of the covariate-dependent detection function (see equation 4 in text).

	Mean	SD	95% CRI
*β_h0_* [Intercept]	1.63	0.37	0.99, 2.46
*β_h1_* [Beaufort sea state]	−0.39	0.10	−0.60, −0.22
*β_h2_* [log(groupSize)]	0.16	0.11	−0.04, 0.40

Coefficients in the table describe the scale parameter (*σ_h,b_*) (equation 4 in text). For a given truncation distance *w* and estimate of *g_b_*(0), a smaller scale parameter (*σ_h,b_*) implies a greater value for *f*(0) and thus a shorter effective strip width and smaller average detection probability (*q_t_*). Thus, for example, the negative coefficient for the linear covariate for Beaufort sea state implies decreasing detectability with worsening (increasing) sea state.

### Abundance and trends based on survey data

Based upon analysis of four separate taxa (i.e., including unidentified ziphiids as a separate taxon), the posterior mean estimates for the trend parameters (*β*_1_) indicated annual rate of change of −4.5% per year (95% CRI: −11.5% to +2.2%) for *Z. cavirostris* and −8.4% (95% CRI: −18.9% to +0.3%) for *Mesoplodon* over the period 1991–2008. The probabilities of declining trend (i.e., Prob[*β*_1_<0]) were 0.92 and 0.97, respectively. No trend was evident for *B. bairdii*, given high uncertainty in parameter estimates (posterior mean trend estimate = +0.8% per year, 95% CRI: −7.6% to +9.8%; probability of decline = 0.44).

**Figure 5:**
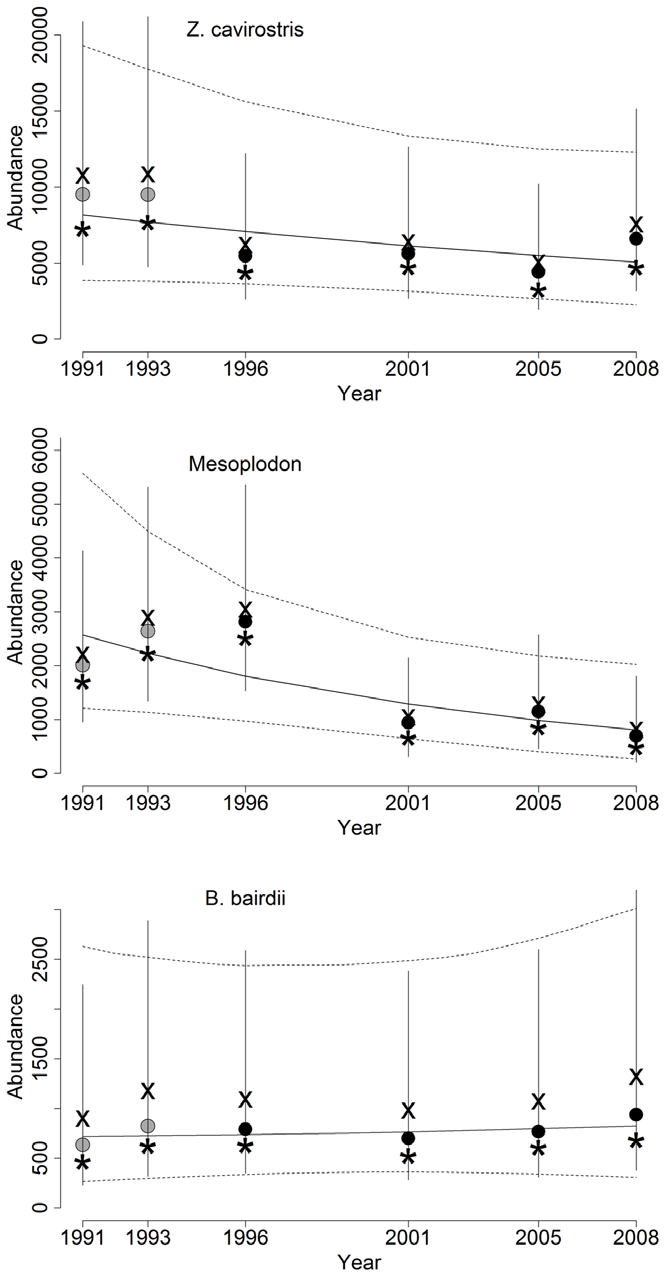
Abundance and trend estimates for beaked whales in the California Current, 1991–2008. *Z. cavirostris* and *Mesoplodon* spp. estimates are pro-rated to include a proportion of the abundance estimated for the “unidentified ziphiid” group. For each year, the Bayesian posterior median (•), mean (X) and mode (*) abundance estimates are shown, along with 90% CRIs. Trend lines depict median and 90% CRI estimates of fitted abundance without process error (e.g., equation 1, with *γ_t_* = 0). Gray points for median estimates in 1991 and 1993 denote that the total abundance estimate for the study area reflects extrapolated density estimates to the Oregon-Washington survey stratum.

The ‘unidentified ziphiid’ group showed evidence of increasing trend (mean trend = +5.0% annually; 95% CRI: −4.8% to +16%), with mean abundance estimates of ≈700 (CV = 0.76) in 1991 and ≈2000 (CV = 0.65) in 2008. One possible explanation for this is that, since observing conditions coincidentally worsened with each survey, there was an increasing trend in the number of sighted groups that could not be identified to genus. Assuming the abundance estimates for unidentified ziphiids comprised a mixture *Z. cavirostris* and *Mesoplodon*, proportionally allocating the estimates to the two species groups and re-estimating the trend parameters weakened the evidence slightly for *Z. cavirostris* and *Mesoplodon* decline. Still, the revised annual growth rate estimate for *Z. cavirostris* was −2.9% per year (95% CRI: −8.8% to +3.3%) and for *Mesoplodon* was −7.0% (95% CRI: −16.7% to +1.0%). The revised estimates for probability of negative trend were 0.84 and 0.96, respectively. Survey abundance estimates that include prorating of individuals from the unidentified ziphiid group to *Z. cavirostris* and *Mesoplodon* are in Fig. 5 and Tables S2, S3, S4.

### Sensitivity analysis

We conducted a *post hoc* analysis to make sure that our trend results for *Z. cavirostris* and *Mesoplodon* were not an artifact of pooling data across all survey strata even though the Oregon-Washington stratum had not been surveyed in 1991 and 1993. Conceivably, if this stratum has lower mean beaked whale densities than the California strata, this could reduce the overall density (and hence abundance) estimates for the 1996–2008 surveys, relative to the 1991 and 1993 survey estimates, leading potentially to a false trend result. Therefore, we repeated the analysis using count (*n_t_*) and effort (*L_t_*) and survey covariate data (e.g. Beaufort state) in all years from only the three California strata to estimate annual average detection rates (*q_t_*), density, and abundance. Data from all strata were still used to estimate the detection function parameters (e.g., model for *σ_h,b_*) and group size. We also included an indicator variable for *B. bairdii* in the detection covariate model, in case the trend estimates were sensitive in any way to how data pooling across species affects the detectability estimates.

This revised analysis did not fundamentally change our inference about trends for any species, including the unidentified group. The probabilities of declining trend for *Z. cavirostris* and *Mesoplodon*, after pro-rating the abundance and trend estimates by those of the unidentified ziphiid group, were 0.86 and 0.96, respectively, virtually identical to those in the primary analysis. Posterior mean estimates for the trend parameters were actually slightly more negative in this *post hoc* analysis (−4.2% and −8.3%, respectively). Given this result, we proceed with discussion based on our primary results, to take advantage of precision and inference from the full dataset.

### Trends in stranding data

**Figure 6:**
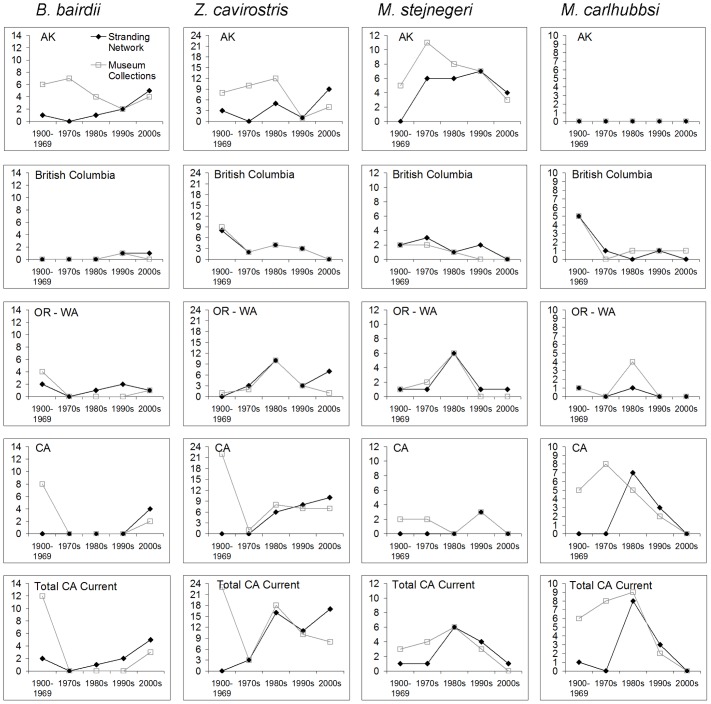
Historical beaked whale stranding records. Number of unique stranding events reported along the west coasts of U.S. and Canada, 1900–2009, for the four most common beaked whale species in the region. Apart from the first data bin (1900–1969), data are binned by decade (e.g., 1970–1979, …, 2000–2009). Data were from two sources: regional stranding networks (black) and museum collections (gray) (Table S1); there is some data redundancy between these two source types. Bottom panel for each species, “Total CA Current”, is the sum of records from OR-WA and CA.

Taken alone, patterns in the historical stranding record provide limited information about beaked whale abundance trends. However, the stranding record appears generally consistent with results of the line-transect survey analyses, thus providing secondary support for our primary analyses. Regional stranding networks originated during the early to mid-1980s, and beach coverage and reporting rates are thought to have increased throughout the 1990s and in to the early 2000s (e.g., [26], [24], pers. comm. with stranding network coordinators). Therefore, for a stable population, an overall increasing trend in stranding reports between the 1980s and 2000s might be expected. The strandings data for *B. bairdii* in the California Current are generally in line with this expectation (Fig. 6). Patterns of *Z. cavirostris* strandings data are highly variable across stranding network regions, but an overall increasing trend from the 1980s through 2000s is not particularly evident across the California Current area, contrary to expectation if the number of reports simply tracked increasing reporting rates. Reports of *Mesoplodon* in the California Current area have clearly decreased over the course of three decades. For *M. carlhubbsi* and *M. stejnegeri*, the decline in reports has been monotonic (when binned by decade) since the 1980s. *M. carlhubbsi* had not been reported along the U.S. coast since 1996 until a report from Washington in 2010 (and none since). *M. perrini* is known as a species from only four stranding events in California, three between 1975 and 1979 and one in 1997. The warm-water species *M. ginkodens*, *M. densirostris*, and *M. peruvianus* are represented by few records within the study area, all from California, the most recent having occurred in 2001 (*Mg*: 1954; *Md*: 1977, 1984, 1985; *Mp*: 1998, 2001).

## Discussion

### Comparison with previous estimates

We compared our estimates with previous estimates [18], [24] obtained using data from the same surveys. The most noteworthy difference is that our estimates were much more stable from year to year. Just as one example, the previous point estimates for *B. bairdii* ranged from 0 to 1591 across years, reflecting strong sensitivity to the small number of groups observed in a particular year, whereas our estimates varied only by a few hundred individuals from year to year (Fig. 5). A key feature of the hierarchical modeling process is to treat the observations as random variables and borrow from the strength of information in the whole dataset to improve individual year estimates, shrinking them more toward the mean trend estimate in more data-poor years and thus improving the precision of each. This, along with taking a probabilistic (Bayesian) approach to inference (sensu [27]), seem to allow for better assessment of population status than would be expected based on guidelines rooted in conventional power analyses. Taylor et al. [10] noted that when using simple regression and a null hypothesis-testing paradigm with significance criteria α = 0.05, even *annual* surveys (for 15 years) of a species would fail half the time to detect a 5% rate of decline when the abundance estimate CV = 0.34. Our annual CVs for *Z. cavirostris* and *Mesoplodon* were substantially higher (0.40–0.65) and the estimated rate of decline for *Z. cavirostris* was less than 5%. Thus, our analysis suggests we can be more optimistic about our ability to assess trends using methods such as those presented here than about using conventional regression-based approaches.

On average, the population abundance estimates for *Z. cavirostris*, *Mesoplodon* (before allocating abundance from the unidentified group) and the group of unidentified ziphiids were higher from our analysis than those reported previously [18], [24]. Specifically, the multi-year average of our posterior median estimates of animal density were 21%, 34%, and 45% higher, respectively, than the average of the earlier point estimates. For *B. bairdii*, the estimates were more similar; the average of our posterior median estimates was 17% lower than theirs, but the average of our posterior mean estimates was 17% higher (because the posterior distribution was right-skewed). Many factors could explain these differences. We used a larger dataset that included observations in Beaufort≤5, whereas the previous analyses were based on observations in Beaufort≤2. Fewer observations in the earlier analyses could have simply introduced higher sampling error. For observations in Beaufort 2, we used a lower estimate of *g*(0) than in the earlier analyses; this would lead to higher abundance estimates in our analysis, all else being equal. If our estimates of *g*(0) were more biased (low) for higher Beaufort states than for Beaufort 0 & 1, this would also lead to higher abundance estimates. In contrast, our slightly lower group size estimates would decrease our abundance estimates. Finally, a suite of differences in how we modeled the detection function (covariates used, species-pooling decisions) could have all affected the results in different ways. Overall, we believe our estimates represent the best estimates to date, given the methods used, the larger data set, and the fuller accounting of Beaufort-dependent *g*(0) estimates.

### Summary of evidence for declining trends

Our analysis suggests that abundance of *Z. cavirostris* and *Mesoplodon* spp. in the California Current has likely declined over the 18-year study period: 1991–2008. The evidence is particularly strong for *Mesoplodon*, based on our analysis of survey-cruise data as well as the strandings record. We cannot say definitively which particular *Mesoplodon* species have driven the observed trend; however, *M. densirostris*, *M. gingkodens*, and *M. peruvianus* are considered tropical or warm-temperate species and are rare in our cold-temperate study area, having been documented in the California Current only from a handful of strandings in southern or central California [15]. Therefore, the declining trend probably does not reflect dynamics of these species but rather of the cold-temperate more abundant species in the California Current: *M. carlhubbsi* (ranges from British Columbia to Baja California), *M. perrini* (known only from stranding records in California), and *M. stejnegeri* (a more northern species; most records are from Alaskan waters with southern California being the southern extent of its known range). Of eight known *Mesoplodon* individuals entangled in the California-based pelagic drift net fishery between 1990 and 1995, five were *M. carlhubbsi*, one was *M. stejnegeri* and two were not identified to species [28].

We do not know whether the observed trends in our study area reflect actual population declines or large-scale distribution shifts to outside of the study area, but there is no obvious evidence for the latter, and given the large study area (ca. 2000 km from north to south boundary), latitudinal distribution shifts would have to be major to substantially reduce the abundance estimates. Abundance increases for long-beaked common dolphins (*Delphinus capensis*) in southern California waters [26], [29] suggest a possible recent northward range shift of warm-water cetacean species in the southern California Current, but warm-water beaked whale species have not been observed in the California stranding record since single events for *M. peruvianus* in 1998 and 2001, and before that since *M. densirostris* in the mid-1980s. For *M. carlhubbsi* and *M. stejnegeri*, the number of reported strandings along the North American west coast peaked in the 1970s or 1980s and has declined more-or-less throughout their respective ranges since then (Fig. 6). In other words, a decline in the number of reports in say, California, does not seem to coincide with increased reports in more northerly areas such as Oregon, Washington, or British Columbia. *Z. cavirostris* stranding numbers in different regions have varied substantially by decade without any obvious pattern suggestive of a latitudinal shift in distribution. We cannot base strong conclusions on the strandings record given the overall rarity of beaked whale occurrences, unquantified variation in stranding-observer effort through time, and diverse sources of variation that can underlie true stranding patterns. We simply make the point that the stranding record does not obviously suggest northward range shifts for beaked whales and seems consistent rather than at-odds with our survey-based abundance analysis.

We can largely rule out sampling design or error artifacts as an explanation for the estimated trends. Seasonal movement dynamics of beaked whales could have changed over the study period such that the timing of our surveys coincided with higher animal abundance in the study area during the first years of the survey; however, beaked whales are not known to migrate, although seasonal movements are poorly understood [7]. Beaked whale identification accuracy by observers could have improved throughout the study period, but decreasing trends in omission error (classifying beaked whale observations as “unidentified”) would imply stronger declining trends than we estimated, because there would have been even more potential beaked whale detections during the earliest surveys. This actually seems possible because the number of “unidentified small whales” (ziphiid spp., minke whale, or *Kogia* sp.) decreased across the course of surveys [18], [24]. Decreasing commission error (e.g., incorrectly labeling an observation as a particular species) seems unlikely at the family level (i.e., non-beaked whales are not likely to have been called beaked whales), while declining error rates at the species or genus level would not change our qualitative inferences, since both *Z. cavirostris* and *Mesoplodon* were estimated to have declined. A final possibility we considered is that visual detectability of beaked whales could have decreased over the course of the study for behavioral reasons or other factors apart from trends in observing conditions. We cannot rule out the possibility of a trend in availability bias (i.e., decreasing *g*(0)); however, preliminary models that included random year effects on *f*(0) suggested that if anything, detection probability of “available” whales may have increased slightly through time after accounting for effects of other covariates. We have no basis for assuming behavioral changes through time related to *g*(0), and such a hypothesis would not explain trends in stranding data. In light of all the evidence, we suggest it is prudent to be precautionary and interpret the apparent declines as real based on the best available information.

### Hypotheses for beaked whale declines

Causes of the apparent declines are unknown, but we consider three hypotheses: effects of incidental mortality from fishing; impacts of anthropogenic noise, namely Navy sonar; and ecosystem changes.

#### Effects of direct mortality from fishing

Bycatch mortality of beaked whales has been reported worldwide, particularly in high-seas driftnet fisheries [30]–[32]; see additional references in [28]. The California large mesh drift gillnet fishery is the only fishery known to interact with beaked whales in the California Current within the US EEZ. Based on U.S. fishery observer program data, annual bycatch mortality estimates from 1990–1995 were 0–6 for *B. bairdii*, 0–44 for *Z. cavirostris*, 0–29 for *Mesoplodon* whales, and 0–15 unidentified ziphiids (based on observer coverage levels of 4.4% to 17.9%; [31]). Comparing the mean 1990–1995 bycatch estimates to our 1991 and 1993 abundance estimates, the mean estimated mortality rate would have been less than 0.005 for both *Z. cavirostris* and *Mesoplodon*. Using the 20^th^ percentile abundance estimates (in line with estimation of Potential Biological Removal under the US Marine Mammal Protection Act), the average bycatch mortality rate would have been as high as 0.008 for *Mesoplodon* in 1991.

Since mid-1996, acoustic pinger deterrents have been used in the California driftnet fishery; this effectively eliminated beaked whale bycatch [33], [28]. A declining trend in fishing effort (Appendix 1 in [16]) and additional regulation of the fishery (reviewed in [34]) – including a large time-area closure (central California to Oregon) in effect for 4 months each year since 2001 to protect leatherback sea turtles (*Dermochelys coriacea*) – have likely reduced the potential for fishery interactions with beaked whales even further.

In summary, it seems unlikely that apparent beaked whale trends in the California Current can be explained by fishery-related mortality inside the US EEZ. Estimated bycatch during the early 1990s appears to have been low relative to abundance estimates, and bycatch of beaked whales since 1996 (inclusive) has presumably been trivial.

#### Navy sonar and other anthropogenic noise

Underwater noise has increased substantially in recent decades [35], and numerous studies and reviews have described the potential and often realized impacts posed to beaked whales and other cetaceans by anthropogenic noise. Of primary concern for beaked whales is noise caused by naval active sonar, although noise associated with varied other sources such as ship traffic and seismic exploration is a potential yet unquantified issue [36]–[42].

*Ziphius* and *Mesoplodon* are the two beaked whale genera known to suffer impacts from naval sonar activities. They exhibit strong behavioral responses to certain types of active sonar, resulting in altered movements and space use for prolonged periods after exposure (e.g., several days; [43], [41]). In more extreme cases there can be physiological consequences leading to death or stranding [44], [45], [37].

Although the threats from naval acoustic activity have been described, population-level impacts have not been quantified. Mass strandings of beaked whales throughout the Northern Hemisphere have been associated with offshore military activity, but estimates of total mortality associated with these types of impacts do not exist. Certainly they exceed levels that have been recorded, however, since the probability of observing dead whales is generally low, especially for deep-water species [46]–[48]. Indirect impacts associated with chronic stress are even more difficult to document, although it could be hypothesized that frequent intense stressors that alter behavior and displace individuals from their habitat could reduce fitness via mechanisms such as reduced foraging efficiency, failed reproduction, increased calf mortality, etc. [42], [49].

Ambient noise off the coast of California has increased many-fold over the past several decades [50], [51], and in the Southern California Bight, beaked whales are exposed to sonar activities in the vicinity of the U.S. Navy's Southern California Anti-Submarine Warfare Range (SOAR). However, evidence to implicate noise from naval activity or other acoustic sources as a cause of apparent beaked whale declines in the California Current is equivocal. If Navy activities at SOAR are responsible, one might expect declines to be localized to the southern California portion of the study area, or even restricted to the SOAR area, depending on beaked whale home range size and movement patterns. Unfortunately, our data do not support a formal evaluation of spatial variation in beaked whale abundance trends; sample sizes from surveys and beach strandings are too low to evaluate abundance trends at a fine spatial scale.

Some Navy ranges support high densities of beaked whales. For example, high densities of *Z. cavirostris* occur in the SOAR area [52], and the Navy's AUTEC sonar test facility in the Tongue of the Ocean (The Bahamas) supports the highest densities of *M. densirostris* that have ever been estimated [53], [54]. High densities are not obviously consistent with a hypothesis that declines are due to military sonar, but they do not refute the possibility that declines have occurred in these areas (i.e., that densities were previously even higher). Navy ranges occurring in high-quality beaked whale habitat could also act as population sinks where sonar-habituated adults persist but recruitment is compromised through direct or indirect mechanisms. Disproportionately high frequencies of immature animals occurring in mass stranding events associated with anthropogenic activities [55] provide some albeit inconclusive support of this hypothesis. Densities of *M. densirostris* in the Abaco Island area, >100 km north of the AUTEC range, appear to have remained stable from 1998–2011 [56], suggesting that, at least for this species in the Bahamas region, any potential negative effects of navy sonar may have a limited geographic reach. However, major differences in deepwater canyon bathymetry and spatial dynamics of naval operations between AUTEC and SOAR (e.g., active sonar operations in the Southern California Bight can occur well outside of SOAR) make it difficult to extend inference for *Mesoplodon* in the Bahamas to *Mesoplodon* and *Z. cavirostris* in the California Current.

#### Ecosystem change

Beaked whale feeding ecology is poorly known. Stomach content analyses from stranded animals suggest that many beaked whale species feed primarily on cephalopods as well as some mid-water and demersal fishes in the deep ocean. *Mesoplodon* whales eat smaller prey and more fish than *Z. cavirostris*, which seems to feed mainly on larger cephalopods [57]. *B. bairdii* feed substantially on demersal fishes, although cephalopods appear important as well [57]–[59].

Dynamics of beaked whale prey are unknown, so it is difficult to infer impacts of ecosystem change on beaked whales in the California Current. However, changes in beaked whale prey in the region may have occurred during our study period. The California Current is a highly variable system, characterized by interannual and interdecadal changes in oceanography (e.g., El Niño, Pacific Decadal Oscillation) that manifest as switches between temperature and associated biological “regimes” [60], [61]. In the middle of our study period (following the strong 1998 El Niño), the California Current switched from a “warm” phase to a “cool” phase. Bottomfish biomass has declined since the 1970s, and by 60% between 2003 and 2010, due in part to effects of demersal fishing [62], [63]. Mesopelagic fishes in the Southern California bight area have declined >60% in abundance since the 1980s in association with increasing deep-water hypoxia due to climate change [64]. Further projected expansion of oxygen minimum zones (OMZ) is expected to have significant ecosystem impacts [65]–[67]. Warming temperatures and OMZ expansion have facilitated invasion of the California Current by jumbo squid (*Dosidicus gigas*) since the late 1990s. *D. gigas* is an abundant, large-bodied (up to 50 kg), aggressive, schooling, hypoxia-tolerant generalist predator of fishes and cephalopods that may have played a role in declines of Pacific hake (*Merluccius productus*) [68], [69], [66]. How deepwater cephalopod communities have responded along with all these factors, either directly or by trophic-mediated pathways, is unknown, but it would be surprising if they have been unaffected. Meanwhile, competition for cephalopod prey between beaked whales and other species could be increasing. Prey species found in stomachs of *M. perrini* and *M. carlhubbsi* in the California Current [70], [71], [58] are also consumed by sperm whales (*Physeter macrocephalus*) [72], jumbo squid [68], and northern elephant seals (*Mirounga angustirostris*) [73], which breed on islands off the coasts of Baja California, Mexico, and southern and central California. Following commercial hunting that depleted elephant seal abundance to only ≈100 by 1890 [74], seals have recovered to over 170,000 as of 2005 [75]. Numbers in U.S. waters have increased roughly 6-fold since the late 1970s and nearly doubled during the course of our study period (M. Lowry, SWFSC, unpubl. data). Ultimately, we lack the information necessary to assess the impacts of ecosystem change and trophic dynamics on beaked whale populations, but additional research into these questions is merited.

### Summary and research recommendations

The abundance of *Ziphius* and especially *Mesoplodon* beaked whales appears to have declined in the California Current since the early 1990s. This inference was made possible through a Bayesian hierarchical modeling approach. Drivers of apparent population declines are unknown, although direct fisheries (bycatch) impacts can probably be ruled out. Impacts from anthropogenic noise and human-mediated or other ecosystem change are plausible explanations, but additional research is required to more thoroughly evaluate these hypotheses.

Dedicated survey effort to estimate trends in the Navy SOAR area of the Southern California Bight and in additional control areas would help test hypotheses concerning the effects of naval sonar on trends. Comparisons of population age structure based on mark-resight data would also be insightful, while data on individual movement patterns would provide complementary information about the potential geographic reach of local impacts at SOAR to other areas of the system. Hypotheses related to ecosystem change could possibly be evaluated through dedicated surveys in areas differently affected by deepwater oxygen depletion, demersal fishing, jumbo squid range expansion, etc., combined with research on spatial or temporal variation in beaked whale diets (e.g., via stomach content and/or stable isotope analysis of free-ranging or stranded animals), prey abundance, and food web modeling. Increased use of acoustic methods to improve the amount of abundance information collected during surveys would be valuable. Additional large-scale surveys (especially augmented by acoustic data collection) will be useful for increasing sample sizes and the length of the time series to eventually permit geographically stratified analysis (ideally in relation to large-scale variation in ecosystem characteristics). This would facilitate our ability to explore factors associated with apparent beaked whale declines.

## Supporting Information

Table S1 **Data sources for beaked whale stranding records from Alaska, British Columbia, Washington, Oregon, and California.** Records in table indicate number of individual animals. These were reduced to the number of unique events (i.e., a group of animals = 1 event) for analysis in main paper
(DOC)

Table S2 **Final abundance estimates (Bayesian posterior summaries) for Cuvier's beaked whale (** ***Ziphius cavirostris*** **) in the California Current study area.** Estimates include pro-rated allocation of unidentified beaked whales to this species.
(DOC)

Table S3 **Final abundance estimates (Bayesian posterior summaries) for** ***Mesoplodon*** **beaked whales in the California Current study area.** Estimates include pro-rated allocation of unidentified beaked whales to this species group.
(DOC)

Table S4 **Final abundance estimates (Bayesian posterior summaries) for Baird's beaked whales (** ***Berardius bairdii*** **) in the California Current study area.**
(DOC)

## References

[1] Committee on Taxonomy (2012) List of marine mammal species and subspecies. Society for Marine Mammalogy. Available: www.marinemammalscience.org. Accessed 2012 May 7.

[2] Baird RW, Webster DL, Schorr GS, McSweeney DJ, Barlow J (2008) Diel variation in beaked whale diving behavior. Marine Mammal Science 24: 630–642.

[3] Hooker SK, Fahlman A, Moore MJ, Aguilar de Soto N, de Quirós YB, et al (2012) Deadly diving? Physiological and behavioural management of decompression stress in diving mammals. Proceedings of the Royal Society B 279: 1041–1050. 22189402 10.1098/rspb.2011.2088 PMC3267154

[4] Pitman R (2009) Mesoplodont whales (*Mesoplodon* spp.). In: Perrin WF, Würsig B, Thewissen JG, editors. Encyclopedia of Marine Mammals, 2^nd^ Ed. Amsterdam: Academic. pp. 721–726.

[5] Davidson AD, Boyer AG, Kim H, Pompa-Mansilla S, Hamilton MJ, et al (2012) Drivers and hotspots of extinction risk in marine mammals. Proceedings of the National Academy of Sciences of the USA 109: 3395–3400. 22308490 10.1073/pnas.1121469109 PMC3295301

[6] IWC (International Whaling Commisssion). (2012a) Review status of Ziphiids in the North Atlantic and Mediterranean Sea. In: Report of the Scientific Committee, Tromsø, Norway, 2011, Annex L: Report of the Sub-Committee on Small Cetaceans. Journal of Cetacean Research and Management 13 (Supplement April 2012): 263–291.

[7] IWC (International Whaling Commission). (2012b) Review status of Ziphiids in the North Pacific and Northern Indian Ocean. In: Report of the Scientific Committee, Panama City, Panama, 2012, Annex L: Report of the Sub-Committee on Small Cetaceans. IWC/64/Rep 1, Annex L, 62 pp.

[8] Taylor BL, Gerrodette T (1993) The uses of statistical power in conservation biology: the vaquita and northern spotted owl. Conservation Biology 7: 489–500.

[9] Thompson W (2004) Sampling Rare or Elusive Species: Concepts, Designs, and Techniques for Estimating Population Parameters. Washington, DC: Island.

[10] Taylor BL, Martinez M, Gerrodette T, Barlow J, Hrovat YN (2007) Lessons from monitoring trends in abundance of marine mammals. Marine Mammal Science 23: 157–175.

[11] Jewell R, Thomas L, Harris CM, Kaschner K, Wiff R, et al (2012) Global analysis of cetacean line-transect surveys: detecting trends in cetacean density. Marine Ecology Progress Series 453: 227–240.

[12] Kéry M, Dorazio RM, Soldaat L, van Strien A, Zuiderwijk A, et al (2009) Trend estimation in populations with imperfect detection. Journal of Applied Ecology 46: 1163–1172.

[13] Kéry M, Royle JA (2010) Hierarchical modelling and estimation of abundance and population trends in metapopulation designs. Journal of Animal Ecology 79: 453–461. 19886893 10.1111/j.1365-2656.2009.01632.x

[14] Moore JE, Barlow JP (2011) Bayesian hierarchical estimation of fin whale abundance trends from a 1991–2008 time series of line-transect surveys in the California Current. Journal of Applied Ecology 48: 1195–1205.

[15] MacLeod CD, Perrin WF, Pitman R, Barlow J, Ballance L, et al (2006) Known and inferred distributions of beaked whale species (Cetacea: Ziphiidae). Journal of Cetacean Research and Management 7: 271–286.

[16] Carretta JV, Forney KA, Oleson E, Martien K, Muto MM, et al.. (2011a) U.S. Pacific Marine Mammal Stock Assessments: 2011. U.S. Department of Commerce, NOAA Technical Memorandum NMFS-SWFSC-488. 356p.

[17] Kinzey D, Olson P, Gerrodette T (2000) Marine mammal data collection procedures on research ship line-transect surveys by the Southwest Fisheries Science Center. NOAA, SWFSC Administrative Report LJ-00-08.

[18] Barlow J, Forney KA (2007) Abundance and the population density of cetaceans in the California Current ecosystem. Fishery Bulletin 105: 509–526.

[19] Buckland ST, Anderson DR, Burnham KP, Laake JL, Borchers DL, et al.. (2001) Introduction to Distance Sampling. New York: Oxford University.

[20] Marques FFC, Buckland ST (2004) Covariate models for the detection function. In: Buckland ST, Anderson DR, Burnham KP, Laake JL, Borchers DL, et al.., editors. Advanced Distance Sampling. New York: Oxford University.

[21] Barlow J (1999) Trackline detection probability for long-diving whales. In: Garner GW, Amstrup SC, Laake JL, Manley BFJ, McDonald LL, et al.., editors. Marine Mammal Survey and Assessment Methods. Rotterdam: Balkema. pp. 209–221.

[22] Lunn DJ, Thomas A, Best N, Spiegelhalter D (2000) WinBUGS – a Bayesian modelling framework: concepts, structure, and extensibility. Statistics and Computing 10: 325–337.

[23] Spiegelhalter DJ, Thomas A, Best N, Lunn D (2007) WinBUGS User Manual, Version 1.4.3, 6 August 2007. Available: http://www.mrc-bsu.cam.ac.uk/bugs/winbugs/contents/shtml. Accessed 2012 Aug 7.

[24] Danil K, Chivers SJ, Henshaw MD, Thieleking JL, Daniels R, et al (2010) Cetacean strandings in San Diego County, California, USA: 1851–2008. Journal of Cetacean Research and Management 11: 163–184.

[25] Barlow J (2010) Cetacean abundance in the California Current estimated from a 2008 ship-based line-transect survey. NOAA Technical Memorandum NOAA-TM-NMFS-SWFSC-456.

[26] Norman SA, Bowlby CE, Brancato MS, Calambokidis J, Duffield D, et al (2004) Cetacean strandings in Oregon and Washington between 1930 and 2002. Journal of Cetacean Research and Management 6: 87–99.

[27] Gerrodette T (2011) Inference without significance: measuring support for hypotheses rather than rejecting them. Marine Ecology – An Evolutionary Perspective 32: 404–418.

[28] Carretta JV, Barlow J, Enriquez L (2008) Acoustic pingers eliminate beaked whale bycatch in a gill net fishery. Marine Mammal Science 24: 956–961.

[29] Carretta JV, Chivers SJ, Perryman WL (2011b) Abundance of the long-beaked common dolphin (*Delphinus capensis*) in the California and western Baja California waters estimated from a 2009 ship-based line-transect survey. Bulletin of the Southern California Academy of Sciences 110: 152–164.

[30] Northridge S (1996) Estimation of cetacean mortality in the U.S. Atlantic swordfish and tuna driftnet and pair-trawl fisheries. NOAA National Marine Fisheries Service, Northeast Fisheries Science Center, Protected Species Division, Woods Hole, MA. Unpublished report.

[31] Julian F, Beeson M (1998) Estimates of marine mammal, turtle, and seabird mortality for two California gillnet fisheries: 1990–1995. Fishery Bulletin 96: 271–284.

[32] Baker CS, Lukoschek V, Lavery S, Dalebout ML, Yong-un M, et al (2006) Incomplete reporting of whale, dolphin and porpoise ‘bycatch’ revealed by molecular monitoring of Korean markets. Animal Conservation 9: 474–482.

[33] Carretta JV, Price T, Petersen D, Read R (2005) Estimates of marine mammal, sea turtle, and seabird mortality in the California drift gillnet fishery for swordfish and thresher shark, 1996–2002. Marine Fisheries Review 66: 21–30.

[34] Moore JE, Wallace B, Lewison R, Zydelis R, Cox T, et al (2009) A review of marine mammal, sea turtle and seabird bycatch in USA fisheries and the role of policy in shaping management. Marine Policy 33: 435–451.

[35] Tyack PL (2008) Implications for marine mammals of large-scale changes in the marine acoustic environment. Journal of Mammalogy 89: 549–558.

[36] Hildebrand JA (2005) Impacts of anthropogenic sound. In: Reynolds III JE, Perrin WF, Reeves RR, Montgomery S, Ragen TJ, editors. Marine mammal research: conservation beyond crisis. Baltimore: Johns Hopkins University. pp. 101–123.

[37] Cox TM, Ragen TJ, Read AJ, Vos E, Baird RW, et al (2006) Understanding the impacts of anthropogenic sound on beaked whales. Journal of Cetacean Research and Management 7: 177–187.

[38] Soto NA, Johnson M, Madsen P, Tyack P, Bocconcelli A, et al (2006) Does intense ship noise disrupt foraging in deep-diving Cuvier's beaked whales (*Ziphius cavirostris*)? Marine Mammal Science 22: 690–699.

[39] Weilgart LS (2007) The impacts of anthropogenic ocean noise on cetaceans and implications for management. Canadian Journal of Zoology 85: 1091–1116.

[40] Parsons ECM, Dolman SJ, Wright AJ, Rose NA, Burns WCG (2008) Navy sonar and cetaceans: just how much does the gun need to smoke before we act? Marine Pollution Bulletin 56: 1248–1257. 18534632 10.1016/j.marpolbul.2008.04.025

[41] Tyack PL, Zimmer WMX, Moretti D, Southall BL, Claridge DE, et al (2011) Beaked whales respond to simulated and actual navy sonar. PloS ONE 6 (3) e17009. 21423729 10.1371/journal.pone.0017009 PMC3056662

[42] Wright AJ, Deak T, Parson ECM (2011) Size matters: management of stress responses and chronic stress in beaked whales and other marine mammals may require larger exclusion zones. Marine Pollution Bulletin 63: 5–9. 20045527 10.1016/j.marpolbul.2009.11.024

[43] McCarthy E, Moretti D, Thomas L, DiMarzio N, Morrissey R, et al (2011) Changes in spatial and temporal distribution and vocal behavior of Blainville's beaked whales (*Mesoplodon densirostris*) during multiship exercises with mid-frequency sonar. Marine Mammal Science 27: E206–E226.

[44] Jepson PD, Arbelo M, Deaville R, Patterson IAP, Castro P, et al (2003) Gas-bubble lesions in stranded animals: Was sonar responsible for a spate of whale deaths after an Atlantic military exercise? Nature 425: 575–76. 14534575 10.1038/425575a

[45] Fernández A, Arbelo M, Deaville R, Patterson IAP, Catsro P, et al (2004) Beaked whales, sonar and decompression sickness. Nature 28 (6984) U1–2. 10.1038/nature02527a 15085881

[46] Faerber MM, Baird RW (2010) Does a lack of observed beaked whale strandings in military exercise areas mean no impacts have occurred? A comparison of stranding and detection probabilities in the Canary and main Hawaiian Islands. Marine Mammal Science 26: 602–613.

[47] Williams R, Gero S, Bejder L, Calambokidis J, Kraus SD, et al (2011) Understanding the damage: interpreting cetacean carcass recoveries in the context of the *Deepwater Horizon*/BP incident. Conservation Letters 4: 228–233.

[48] Perrin WF, Thieleking JL, Walker WA, Archer FI, Robertson KM (2011) Common bottlenose dolphins (Tursiops truncatus) in California waters: Cranial differentiation of coastal and offshore ecotypes. Marine Mammal Science 769–792.

[49] Wright AJ, Soto NA, Bateson M, Beale CM, Clark C, et al (2007) Do marine mammals experience stress related to anthropogenic noise? International Journal of Comparative Psychology 20: 274–316.

[50] Andrew RK, Howe BM, Mercer JA (2002) Ocean ambient sound: comparing the 1960s with the 1990s for a receiver off the California coast. Acoustic Research Letters Online 3: 65–70.

[51] McDonald MA, Hildebrand JA, Wiggins SM (2006) Increases in deep ocean ambient noise in the Northeast Pacific west of San Nicolas Island, California. Journal of the Acoustical Society of America 120: 711–718. 16938959 10.1121/1.2216565

[52] Falcone EA, Schorr GS, Douglas AB, Calambokidis J, Henderson E, et al (2009) Sighting characteristics and photo-identification of Cuvier's beaked whales (*Ziphius cavirostris*) near San Clemente Island, California: a key area for beaked whales and the military? Marine Biology 156: 2631–2640. 24391238 10.1007/s00227-009-1289-8 PMC3873046

[53] Moretti D, DiMarzio N, Morrissey R, Ward J, Jarvis S (2006) Estimating the density of Blainville's beaked whale (*Mesoplodon densirostris*) in the Tongue of the Ocean (TOTO) using passive acoustics, Proceedings of the Oceans 2006 MTS/IEEE Conference, Boston, Massachusetts.

[54] Marques TA, Thomas L, Ward J, DiMarzio N, Tyack PL (2009) Estimating cetacean population density using fixed passive acoustics sensors: an example with beaked whales. Journal of the Acoustical Society of America 125: 1982–1994. 19354374 10.1121/1.3089590

[55] MacLeod CD, D'Amico A (2006) A review of beaked whale behaviour and ecology in relation to assessing and mitigation impacts of anthropogenic noise. Journal of Cetacean Research and Management 7: 211–221.

[56] Claridge DE, Durban JW (2012) Distribution, abundance and population structuring of beaked whales in the Great Bahama Canyon. 2012 Marine Mammals & Biological Oceanography Program Review, Office of Naval Research, Alexandria, Virginia.

[57] Walker WA, Mead JG, Brownell RL Jr (2002) Diets of Baird's beaked whales, *Berardius bairdii*, in the southern Sea of Okhotsk and off the Pacific coast of Honshu, Japan. Marine Mammal Science 18: 902–919.

[58] MacLeod CD, Santos MB, Pierce GJ (2003) Review of data on diets of beaked whales: evidence of niche separation and geographic segregation. Journal of the Marine Biological Association of the U.K. 83: 651–665.

[59] Ohizumi H, Isoda T, Kishiro T, Kato H (2003) Feeding habits of Baird's beaked whale *Berardius bairdii*, in the western North Pacific and Sea of Okhotsk off Japan. Fisheries Science 69: 11–20.

[60] Chavez FP, Ryan J, Lluch-Cota SE, Ñiquen CM (2003) From anchovies to sardines and back: multidecadal change in the Pacific Ocean. Science 299: 217–221. 12522241 10.1126/science.1075880

[61] Peterson WT, Schwing FB (2003) A new climate regime in northeast Pacific ecosystems. Geophysical Research Letters 30: 1896.

[62] Levin PS, Holmes EE, Piner KR, Harvey CJ (2006) Shifts in a Pacific Ocean fish assemblage: the potential influence of exploitation. Conservation Biology 20: 1181–1190. 16922234 10.1111/j.1523-1739.2006.00400.x

[63] Keller AA, Wallace JR, Horness BH, Hamel OS, Stewart IJ (2012) Variations in eastern North Pacific demersal fish biomass based on the U.S. west coast groundfish bottom trawl survey (2003–2010). Fishery Bulletin 110: 205–222.

[64] Koslow JA, Goericke R, Lara-Lopez A, Watson W (2011) Impact of declining intermediate-water oxygen on deepwater fishes in the California Current. Marine Ecology Progress Series 436: 207–218.

[65] Bograd SJ, Castro CG, Di Lorenzo ED, Palacios DM, Bailey H, et al (2008) Oxygen declines and the shoaling of the hypoxic boundary in the California Current. Geophysical Research Letters 35: L12607.

[66] Seibel BA (2011) Critical oxygen levels and metabolic suppression in oceanic oxygen minimum zones. Journal of Experimental Biology 214: 326–336. 21177952 10.1242/jeb.049171

[67] Pierce SD, Barth JA, Shearman RK, Erofeev AY (2012) Declining oxygen in the Northeast Pacific. Journal of Physical Oceanography 42: 495–501.

[68] Field JC, Baltz K, Phillips AJ, Walker WA (2007) Range expansion and trophic interactions of the jumbo squid, *Dosidicus gigas*, in the California Current. CalCOFI Report 48: 131–146.

[69] Zeidberg LD, Robison B (2007) Invasive range expansion by the Humboldt squid, *Dosidicus gigas*, in the eastern North Pacific. Proceedings of the National Academy of Sciences of the USA 104: 12948–12950. 17646649 10.1073/pnas.0702043104 PMC1937572

[70] Mead J (1981) First records of *Mesoplodon hectori* (Ziphiidae) from the Northern Hemisphere and a description of the adult male. Journal of Mammalogy 62: 430–432.

[71] Dalebout ML, Mead JG, Baker CS, Baker AN, van Helden AL (2002) A new species of beaked whale *Mesoplodon perrini* sp. N. (Cetacean: Ziphiidae) discovered through phylogenetic analyses of mitochondrial DNA sequences. Marine Mammal Science 18: 577–608.

[72] Fiscus CH, Rice DW, Wolman AA (1989) Cephalopods from the stomachs of sperm whales taken off California. U.S. Department of Commerce, NOAA Technical Report NMFS 83. Available: National Technical Information Service, 5285 Port Royal Road, Springfield, VA 22161, or http://spo.nwr.noaa.gov/tr83opt.pdf. Accessed 19 July 2012.

[73] Antonelis GA, Lowry MS, Fiscus CH, Stewart BS, DeLong RL (1994) Diet of the northern elephant seal. In: Le Boeuf BJ, Laws RM, editors. Elephant seals: population ecology, behavior, and physiology. Berkeley: University of California Press. pp. 211–223.

[74] Stewart BS, Yochem PK, Huber HR, DeLong RL, Jameson RJ, et al.. (1994) History and present status of the northern elephant seal population. In: Le Boeuf BJ, Laws RM, editors. Elephant seals: population ecology, behavior, and physiology. Berkeley: University of California. pp. 29–48.

[75] Condit R, Lowry MS, Betcher A, Allen SG, Lee DE, et al (2012) Population Status of Northern Elephant Seals. Marine Mammal Science In press.

