# Supplementary material for: Declining Abundance of Beaked Whales (Family Ziphiidae) in the California Current Large Marine Ecosystem
Source: PLoS One. 2013 Jan 16;8(1):e52770. doi: 10.1371/journal.pone.0052770 (PMC3547055; doi:10.1371/journal.pone.0052770)
Supplement: Table S1 — Data sources for beaked whale stranding records from Alaska, British Columbia, Washington, Oregon, and California. Records in table indicate number of individual animals. These were reduced to the number of unique events (i.e., a group of animals = 1 event) for analysis in main paper (DOC) [file pone.0052770.s001.doc]

| Table S1. Data sources for beaked whale stranding records from Alaska, British Columbia, Washington, Oregon, and California. Records in table indicate number of individual animals. These were reduced to the number of unique events (i.e., a group of animals = 1 event) for analysis in main paper | | | |
| --- | --- | --- | --- |
|  | Total no. records | Record date range | Records per species |
| **Stranding Networks** |  |  |  |
| Alaska regional stranding network | 78 | 1947 - 2012 | 9 Berardius; 24 Ziphius; 45 M. stejnegeri |
| British Columbia regional sightings network | 36 | 1900 - 2010 | 2 Berardius; 20 Ziphius; 6 M. carlhubbsi; 8 M. stejnegeri |
| Northwest regional stranding network | 43 | 1942 - 2010 | 6 Berardius; 23 Ziphius; 3 M. carlhubbsi; 11 M. stejnegeri |
| California regional stranding network | 47 | 1982 - 2010 | 4 Berardius; 25 Ziphius; 11 M. carlhubbsi; 4 M. stejnegeri; 2 M. densirostris; 1 M. perrini |
| **Museum Collections** |  |  |  |
| Smithsonian (NMNH)* | 221 | 1902 - 2010 | 33 Berardius; 95 Ziphius; 24 M. carlhubbsi; 63 M. stejnegeri; 4 M. perrini; 1 M. peruvianus; 1 M. ginkogdens |
| Los Angeles County Museum of Natural History (LACM) | 34 | 1907 - 2007 | 2 Berardius; 18 Ziphius; 5 M. carlhubbsi; 5 M. stejnegeri; 2 M. perrini; 2 M. peruvianus |
| U. of Alaska Museum (UAM) | 16 | 1957 - 2009 | 2 Berardius; 6 Ziphius; 8 M. stejnegeri |
| California Academy of Sciences, Kimball Natural History Museum (CAS) | 16 | 1925 - 2003 | 2 Berardius; 4 Ziphius; 5 M. carlhubbsi; 2 M. stejnegeri; 3 M. densirostris |
| Berkeley Museum of Vertebrate Zoology (MVZ) | 8 | 1952 - 2001 | 2 Berardius; 2 Ziphius; 3 M. carlhubbsi; 1 M densirostris |
| U Washington Burke Museum | 6 | 1950 - 1987 | 1 Berardius; 1 Ziphius; 1 M. carlhubbsi; 3 M. stejnegeri |
| Santa Barbara Museum of Natural History (SBNHM) | 6 | 1984 - 2010 | 3 Ziphius; 2 M. carlhubbsi; 1 M. densirostris |
| San Diego Natural History Museum (SDNHM) | 4 | 1950 - 1980 | 3 Ziphius; 1 M. peruvianus |
| Chicago Field Museum (FMNH) | 1 | 1964 | 1 Ziphius |
| * There are some duplicate records between Smithsonian and other museums; these were removed for analysis presented in main paper  Links to data sources:   | http://www.nmfs.noaa.gov/pr/health/networks.htm | NOAA - marine mammal stranding networks | | --- | --- | | http://wildwhales.org/ | British Columbia cetacean sightings network | | http://arctos.database.museum/home.cfm | Arctos data portal | | http://manisnet.org/portals.html | MaNIS data portal | | http://collections.nmnh.si.edu/search/mammals/ | Smithsonian museum, mammals collection | | | | |
