# Supplementary material for: Declining Abundance of Beaked Whales (Family Ziphiidae) in the California Current Large Marine Ecosystem
Source: PLoS One. 2013 Jan 16;8(1):e52770. doi: 10.1371/journal.pone.0052770 (PMC3547055; doi:10.1371/journal.pone.0052770)
Supplement: Table S2 — Final abundance estimates (Bayesian posterior summaries) for Cuvier's beaked whale ( Ziphius cavirostris ) in the California Current study area. Estimates include pro-rated allocation of unidentified beaked whales to this species. (DOC) [file pone.0052770.s002.doc]

| Table S2. Final abundance estimates (Bayesian posterior summaries) for Cuvier’s beaked whale (*Ziphius cavirostris*) in the California Current study area. Estimates include pro-rated allocation of unidentified beaked whales to this species. | | | | | | |
| --- | --- | --- | --- | --- | --- | --- |
| Year | Mode | Median | Mean | SD | CV | 90% CRI |
| 1991 | 7241 | 9509 | 10771 | 5488 | 0.51 | 4893 – 20880 |
| 1993 | 7667 | 9504 | 10843 | 5875 | 0.54 | 4751 – 21220 |
| 1996 | 4392 | 5460 | 6204 | 3359 | 0.54 | 2615 – 12220 |
| 2001 | 4720 | 5607 | 6381 | 3460 | 0.54 | 2661 – 12656 |
| 2005 | 3233 | 4416 | 5046 | 2861 | 0.57 | 1941 – 10226 |
| 2008 | 4701 | 6590 | 7550 | 4173 | 0.55 | 3158 – 15150 |
