# Supplementary material for: Declining Abundance of Beaked Whales (Family Ziphiidae) in the California Current Large Marine Ecosystem
Source: PLoS One. 2013 Jan 16;8(1):e52770. doi: 10.1371/journal.pone.0052770 (PMC3547055; doi:10.1371/journal.pone.0052770)
Supplement: Table S3 — Final abundance estimates (Bayesian posterior summaries) for Mesoplodon beaked whales in the California Current study area. Estimates include pro-rated allocation of unidentified beaked whales to this species group. (DOC) [file pone.0052770.s003.doc]

| Table S3. Final abundance estimates (Bayesian posterior summaries) for *Mesoplodon* beaked whales in the California Current study area. Estimates include pro-rated allocation of unidentified beaked whales to this species group. | | | | | | |
| --- | --- | --- | --- | --- | --- | --- |
| Year | Mode | Median | Mean | SD | CV | 90% CRI |
| 1991 | 1700 | 2009 | 2206 | 1026 | 0.46 | 952 – 4135 |
| 1993 | 2223 | 2639 | 2895 | 1297 | 0.45 | 1339 – 5313 |
| 1996 | 2509 | 2811 | 3045 | 1233 | 0.41 | 1533 – 5360 |
| 2001 | 656 | 939 | 1047 | 592 | 0.57 | 311 – 2144 |
| 2005 | 851 | 1144 | 1280 | 683 | 0.53 | 459 – 2571 |
| 2008 | 481 | 694 | 811 | 528 | 0.65 | 204 – 1807 |
