# Supplementary material for: Declining Abundance of Beaked Whales (Family Ziphiidae) in the California Current Large Marine Ecosystem
Source: PLoS One. 2013 Jan 16;8(1):e52770. doi: 10.1371/journal.pone.0052770 (PMC3547055; doi:10.1371/journal.pone.0052770)
Supplement: Table S4 — Final abundance estimates (Bayesian posterior summaries) for Baird's beaked whales ( Berardius bairdii ) in the California Current study area. (DOC) [file pone.0052770.s004.doc]

| Table S4. Final abundance estimates (Bayesian posterior summaries) for Baird’s beaked whales (*Berardius bairdii*) in the California Current study area | | | | | | |
| --- | --- | --- | --- | --- | --- | --- |
| Year | Mode | Median | Mean | SD | CV | 90% CRI |
| 1991 | 464 | 635 | 900 | 1228 | 1.37 | 229 – 2241 |
| 1993 | 623 | 821 | 1176 | 1570 | 1.33 | 318 – 2888 |
| 1996 | 629 | 790 | 1092 | 1376 | 1.26 | 349 – 2589 |
| 2001 | 522 | 699 | 981 | 1260 | 1.28 | 282 – 2382 |
| 2005 | 606 | 767 | 1072 | 1384 | 1.29 | 309 – 2599 |
| 2008 | 682 | 937 | 1320 | 1768 | 1.34 | 378 – 3196 |
